# Comparative lipidomic analysis of mammalian retinal ganglion cells and Müller glia in situ and in vitro using High-Resolution Imaging Mass Spectrometry

**DOI:** 10.1038/s41598-020-77087-x

**Published:** 2020-11-18

**Authors:** Xandra Pereiro, Roberto Fernández, Gabriel Barreda-Gómez, Noelia Ruzafa, Arantxa Acera, Javier Araiz, Egoitz Astigarraga, Elena Vecino

**Affiliations:** 1 Experimental Ophthalmic-Biology Group (GOBE), Department of Cell Biology and Histology, University of Basque Country (UPV/EHU), Leioa, Vizcaya Spain; 2 Department of Physical Chemistry, University of the Basque Country (UPV/EHU), Leioa, Vizcaya Spain; 3 IMG Pharma, Derio, Vizcaya Spain; 4 Department Ophthalmology, University of the Basque Country (UPV/EHU), Leioa, Vizcaya Spain

**Keywords:** Cellular imaging, Cellular neuroscience

## Abstract

In order to better understand retinal physiology, alterations to which underlie some ocular diseases, we set out to establish the lipid signature of two fundamental cell types in the retina, Müller Glia and Retinal Ganglion Cells (RGCs). Moreover, we compared the lipid signature of these cells in sections (in situ), as well as after culturing the cells and isolating their cell membranes (in vitro). The lipidome of Müller glia and RGCs was analyzed in porcine retinal sections using Matrix Assisted Laser Desorption Ionization Imaging Mass Spectrometry (MALDI-IMS). Isolated membranes, as well as whole cells from primary cell cultures of RGCs and Müller glia, were printed onto glass slides using a non-contact microarrayer (Nano Plotter), and a LTQ-Orbitrap XL analyzer was used to scan the samples in negative ion mode, thereafter identifying the RGCs and Müller cells immunohistochemically. The spectra acquired were aligned and normalized against the total ion current, and a statistical analysis was carried out to select the lipids specific to each cell type in the retinal sections and microarrays. The peaks of interest were identified by MS/MS analysis. A cluster analysis of the MS spectra obtained from the retinal sections identified regions containing RGCs and Müller glia, as confirmed by immunohistochemistry in the same sections. The relative density of certain lipids differed significantly (p-value ≤ 0.05) between the areas containing Müller glia and RGCs. Likewise, different densities of lipids were evident between the RGC and Müller glia cultures in vitro. Finally, a comparative analysis of the lipid profiles in the retinal sections and microarrays identified six peaks that corresponded to a collection of 10 lipids characteristic of retinal cells. These lipids were identified by MS/MS. The analyses performed on the RGC layer of the retina, on RGCs in culture and using cell membrane microarrays of RGCs indicate that the lipid composition of the retina detected in sections is preserved in primary cell cultures. Specific lipid species were found in RGCs and Müller glia, allowing both cell types to be identified by a lipid fingerprint. Further studies into these specific lipids and of their behavior in pathological conditions may well help identify novel therapeutic targets for ocular diseases.

## Introduction

The retina is a light-sensitive multi-layered tissue that lines the back of the eye and that is responsible for converting light into an electrical signal. Interactions between retinal glial cells and the neurons that communicate between the eye and the brain, the retinal ganglion cells (RGCs), are essential for the retina to function correctly. Müller glia are the most abundant glia in the retina, constituting 90% of the retinal glia. They are radially oriented cells that extend from the inner (vitreal) border of the retina to the distal end of the outer nuclear layer (ONL). These are specialized radial glial cells that establish a physical and functional link between the neurons and their environment, a milieu that contains blood vessels, the vitreous humor and the sub-retinal space. These glia play a role in maintaining the structural integrity of the retina and they sustain retinal homeostasis by participating in essential processes like glucose metabolism, substrate exchange and vascular regulation. RGCs are the output neurons in the retina and it is their axons that form the optic nerve that carries the electrical signals from the retina to the visual centers in the brain^1,2^.

Lipids are fundamental constituents of the central nervous system (CNS), and defective lipid metabolism is related to a number of diseases of the brain and peripheral nervous system (PNS)^3^. The activity of cells throughout the body is regulated by their plasma membrane, in which planar lipid microdomains known as “lipid rafts”^4^ constitute dynamic platforms for multiple cell signaling events^5^, including the signaling that controls cell adhesion, cell migration, inflammation or immune reactions^6^. There are many unique lipids in the retina that play a fundamental role in retinal function and disease. For example, Polyunsaturated Fatty Acids (PUFAs) with Docosahexaenoic acid (DHA, 22:6, 22 carbons with 6 double bonds) are representative of the retina, accounting for approximately 50% of the fatty acids in photoreceptors^7^. This accumulation of such large amounts of DHA in retinal membranes makes them very fluid, favoring efficient conformational changes during phototransduction. Moreover, the DHA-derived mediator neuroprotectin D1, is also involved in protective, anti-inflammatory and pro-survival reparative signaling^8–12^.

Cholesterol is another important retinal lipid and indeed, it is the second most abundant lipid in the neuroretina behind phospholipids^13^. Free cholesterol plays key roles in the modulation of vesicle cycling, ion channels and dendritic spine development^6,14,15^, and the Müller glia in the retina represent the principal hub for the de novo production and transport of cholesterol^16,17^. The access of Müller glia to all the neurons and other glial cells in the retina that form the neurogliovascular unit enables them to control the transport of ions, water, lipids and protein across the inner blood-retina barrier^18,19^. Thus, manipulating the lipid microenvironment affects the interpretation of different retinal stimuli by these glia (chemical, osmotic and temperature), potentially contributing to retinal pathologies^20^. In fact, altered cholesterol levels underlie debilitating, blinding neurodegenerative diseases like Smith-Lemli-Opitz and Niemann-Pick Syndromes^21^. It has also been shown that over and underexposure to cholesterol can contribute to the progression of several multifactorial diseases like glaucoma, diabetic retinopathy and macular edema, modulating Müller glial sensing and the transduction of ambient information in the retina. Significantly, animals fed cholesterol-deprived or cholesterol-enriched diets suffer a loss of neurons^6,16,19,22^. A lipid shuttle has been identified from Müller glia to neurons that covers the neurons’ lipid demands, especially those related to the maintenance/renewal of the long projection axons of RGCs and to synaptogenesis^18,23^. Hence, it is clearly important to understand the behavior of lipids in the retina, and specifically in relation to the Müller glia and RGC dynamics.

Matrix Assisted Laser Desorption Ionization Imaging Mass Spectrometry (MALDI-IMS) is a technique that combines mass spectrometry with histology. MALDI-IMS technology has progressed rapidly over the past decade, with significant improvements in instrumentation^24,25^, laser technology^26^, sample preparation^27,28^ and bioinformatics analysis. These advances have enhanced the sensitivity of the technique, lowering the acquisition time and providing greater spatial resolution, together broadening the type of tissue, samples and features that can be analyzed^29^. The distribution of retinal lipids has been studied previously by MALDI-IMS in mouse^30^, salamander^31^ and pig tissues^32^, and in human^33^ tissue sections. Retinal lipids from rat and human retinal tissue have been studied in negative ion mode by combining chloroform/methanol extraction of homogenized tissue with liquid chromatography (LC)–MS^34^. However, no information on the spatial distribution of these lipids could be gained in this way, nor of their cell type specificity in the retina. The spatial distribution and identity of lipid and retinoid metabolites are characteristic of specific retinal cell layers^31,33,35,36^, as seen in Abca4^–/–^ mice (a model of Stargardt disease) when analyzed using high spatial resolution MALDI-IMS. However, these studies mainly focused on the lipids in the photoreceptor cell layers (the Outer segment and ONL) and the retinal pigment epithelium (RPE)^36^.

Thus, and despite their importance as causative and diagnostic molecules for retinal disease, there is little known about the lipid profiles in the normal retina and the differences in the lipid composition of the cells that make up the retina. To address this issue, we set out here to identify the lipid signature of Müller glia and RGCs in sections of the porcine retina, and in primary cell cultures, using MALDI-IMS. We also generated whole cell and cell membrane microarrays^36^ from cultures of RGCs and Müller glia, comparing the results obtained to the lipids observed in retinal sections. The reproducibility of these microarrays is associated with reduced noise and their sensitivity provides an accurate measure of the relative intensity of the lipids analyzed^37^. We used the porcine retina due to its similarity in size and structure to the human retina^38–40^. Indeed, our model of glaucoma in pig displays a same pattern of neuron degeneration as that seen in humans^41^.

## Results

**Figure 1:**
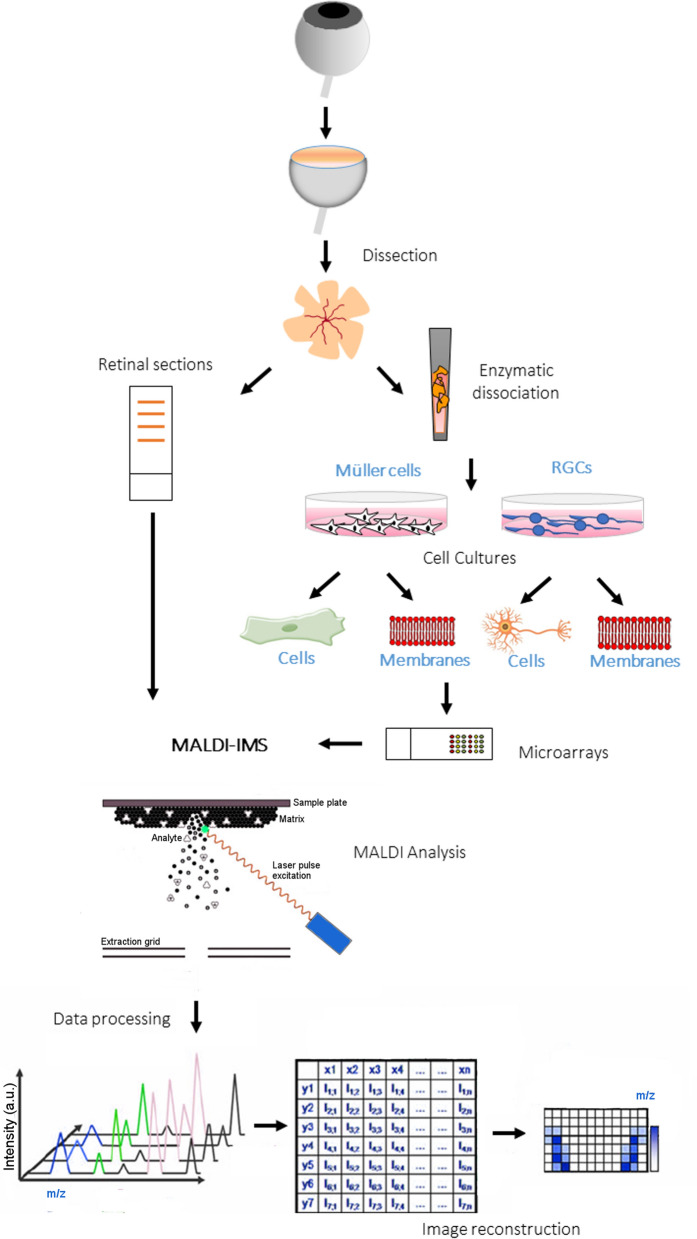
Lipidomics work flow. Scheme followed to identify the lipid signature in retinal sections, that of RGC and Müller cell cultures, and that of the cell membranes isolated from both these cultures.

**Figure 2:**
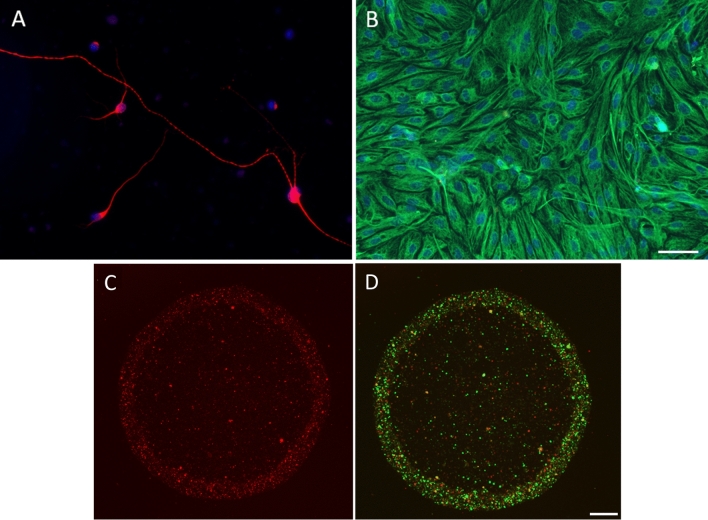
Representative images of pig retinal cultures. (**A**) RGC stained with Beta III tubulin antibody (red), (**B**) pure Müller cells culture stained with the vimentin antibody (green) and DAPI used as nuclear marker in blue. (**C**) Immunohistochemical analysis of cells, with RGCs stained with the Beta III tubulin antibody (red). (**D**) Membranes from Müller cells culture stained with a p75 antibody (green). Scale bars: 50 μm.

In order to identify a lipid signature for RGCs and Müller cells, a strategy was developed that integrates in situ and in vitro experiments within a lipidomics workflow (Fig. 1). Prior to performing the lipidomic analysis, the purity and quality of the cultures established was verified by immunocytochemistry using antibodies that specifically recognized RGCs and Müller glia. After 7 days in vitro (div), RGCs had grown and on the whole, regenerated their neurites (Fig. 2A), while Müller cell cultures were confluent (Fig. 2B). Moreover, the Müller cells maintained their viability and normal function, expressing specific markers like p75NTR, CRALBP and Glutamine synthetase (Supplementary Fig. 1).

The data from the MS spectral analysis of the microarrays established from the Müller cell and RGC cultures were compared, highlighting significant differences in the relative lipid densities between these two cell types. Two different microarrays were prepared from the cultures for the MS spectral analysis, one from whole cells and the other from membrane preparations. The MS spectral analysis highlighted the differential negative ions (m/z) and the normalized relative intensities in whole cells or membrane preparations of the cultured RGCs and Müller cells (Table 1). Similar results were obtained for the whole cell and membrane preparations for each cell type, highlighting the consistency of the data.

**Table 1:** Summary of the differential negative ions (*m/z*), and the normalized relative intensity obtained after MS spectral analysis of the microarrays prepared from whole cell or membrane preparations of RGC and Müller cell cultures.

Ion	Intensity				
	Membranes			Cells	
	Müller	RGC		Müller	RGC
599,32	9 ± 2	35 ± 4		1 ± 1	11 ± 3
700,52	19 ± 3	41 ± 2		5 ± 3	21 ± 8
716,52	49 ± 2	133 ± 4		34 ± 10	162 ± 16
722,51	28 ± 5	69 ± 5		10 ± 10	43 ± 18
726,54	5 ± 0	9 ± 1		1 ± 0	7 ± 0
738,50	3 ± 1	15 ± 1		0 ± 1	10 ± 1
740,52	7 ± 1	11 ± 2		2 ± 1	11 ± 2
742,54	61 ± 6	150 ± 10		42 ± 17	158 ± 15
746,57	67 ± 5	11 ± 2		15 ± 8	5 ± 1
764,52	7 ± 2	70 ± 5		2 ± 2	57 ± 10
770,57	59 ± 7	66 ± 4		45 ± 17	124 ± 13
771,64	9 ± 2	11 ± 3		3 ± 0	14 ± 1
772,58	274 ± 20	35 ± 2		200 ± 81	70 ± 11
774,52	8 ± 2	3 ± 3		8 ± 0	7 ± 1
776,56	26 ± 3	42 ± 3		13 ± 8	33 ± 11
790,54	278 ± 22	211 ± 12		159 ± 65	165 ± 21
794,57	77 ± 9	37 ± 1		54 ± 21	52 ± 6
797,65	11 ± 1	49 ± 5		6 ± 1	81 ± 5
834,53	8 ± 4	73 ± 7		0 ± 1	4 ± 1
836,53	1 ± 0	20 ± 3		0 ± 1	4 ± 2
863,56	4 ± 0	50 ± 4		0 ± 0	9 ± 0
885,55	622 ± 100	938 ± 40		103 ± 55	254 ± 54
909,55	20 ± 3	74 ± 5		3 ± 2	21 ± 4
913,58	5 ± 1	14 ± 3		0 ± 1	4 ± 3

**Figure 3:**
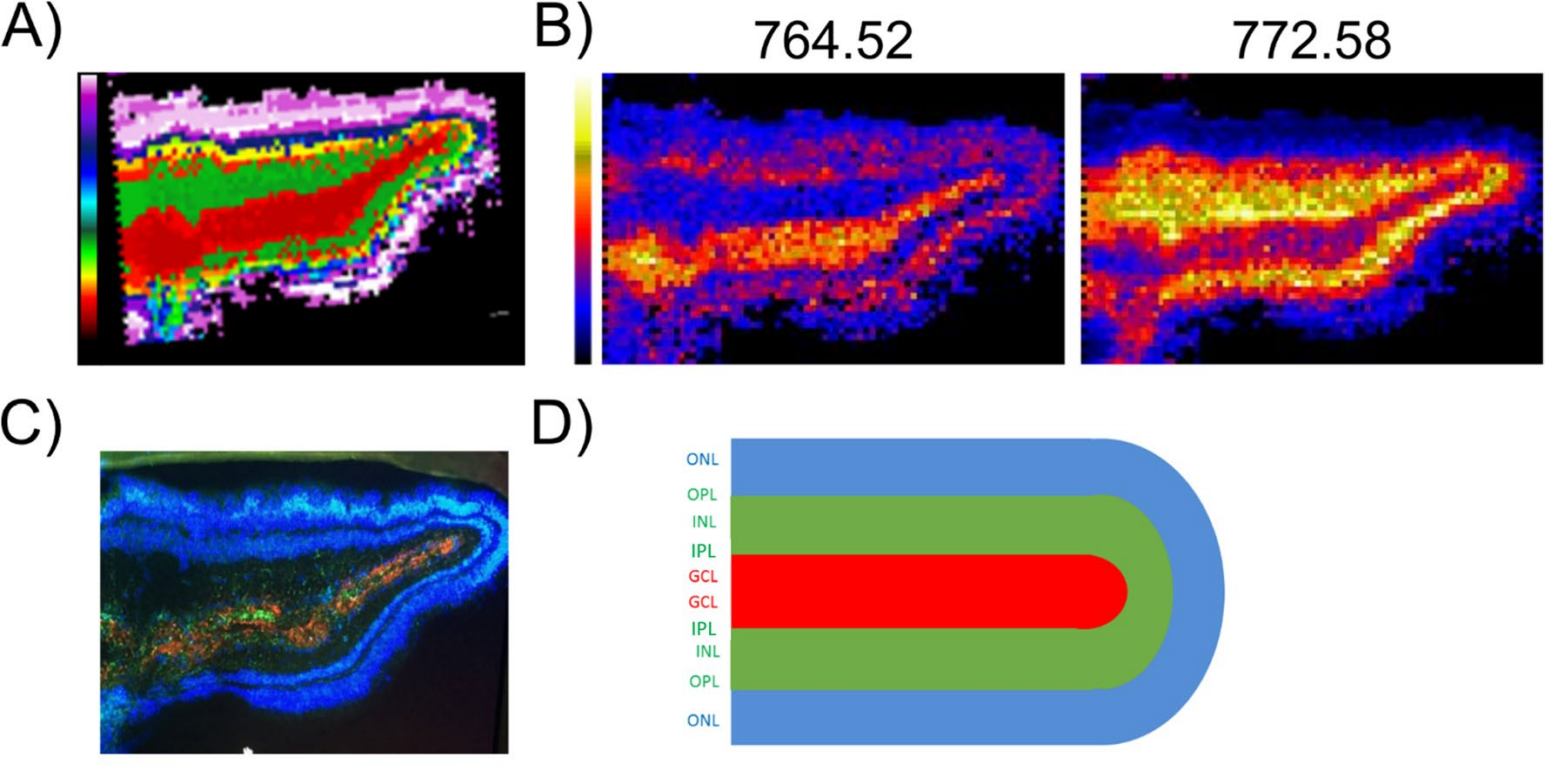
IMS-MALDI analysis of retinal sections. (**A**) Divisive Hierarchical Clustering based on the correlation distance, the colors of the clusters in the bar indicate the distance (1-correlation) between the average spectra of the clusters. (**B**) Two examples of peaks *m/z* 764.52 and 772.58 that correspond to areas containing RGCs (GCL and IPL) or Müller cells (INL and OPL). (**C**) Immunohistochemical analysis of the retinal section previously analyzed by MALDI-IMS, with the RGCs labeled with the Beta III tubulin antibody (red), Müller cells labeled with the vimentin antibody (green) and nuclei stained in blue (DAPI) in a previously scanned retinal section. (**D**) Scheme showing the layer arrangement of the retinal sections. Nerve fiber layer (NFL), ganglion cell layer (GCL), inner plexiform layer (IPL), inner nuclear layer (INL), outer plexiform layer (OPL), outer nuclear layer (ONL).

Following the MALDI-IMS analysis of retinal sections, a cluster analysis of the MS spectra identified regions that corresponded to areas of RGCs and Müller glia, as confirmed by immunohistochemistry of these previously scanned sections (Fig. 3). The cluster analysis successfully identified the area that corresponded to RGCs (the ganglion cell layer, GCL) and the rest of the layers in the retina. Müller cells span the entire thickness of the retina, sharing their space with other cells and with RGCs. Therefore, the GCL area that corresponded to the RGC population (red) was compared with the area where Müller cells overlap with other cell bodies, the area that mostly corresponds to the inner plexiform layer (IPL, green). The raw spectra obtained from both these clusters or areas can be seen in the supplementary information (Supplementary Data 1). There was a significant difference in the relative density of certain lipids identified in the MS/MS analysis between areas containing RGCs and those of Müller glia (p-value ≤ 0.05, Table 2).

**Table 2:** Summary of the differential negative ions (*m/z*) and the corresponding lipid species, together with the relative intensity, determined in a MALDI-IMS analysis of the Müller glia and RGC domains of retinal sections.

Ion	Lipid molecular species	Intensity		
		Müller		RGC
599,32	Lyso-PI 18:0; PI O-16:0/2:0	47 ± 9		64 ± 9*
700,52	PE P-16:0/18:1; PE O-16:1/18:1	56 ± 10		99 ± 20**
716,52	PC 16:1/16:0; PC 16:0/16:1 PE 18:1/16:0; PE 16:0/18:1 PE 18:0/16:1	120 ± 14		171 ± 24*
722,51	PE P-16:0/20:4	100 ± 29		163 ± 42*
726,54	PE O-18:2/18:1; PE P-18:1/18:1	15 ± 5		33 ± 9*
738,50	PE 16:0/20:4	19 ± 4		35 ± 11*
740,52	PE 18:1/18:2	10 ± 3		19 ± 4*
742,54	PC 18:2/16:0; PE 18:1/18:1	110 ± 15		199 ± 19***
746,57	PC 18:0/16:0; PE 18:0/18:0	153 ± 45		83 ± 21*
764,52	PE 18:1/20:4; PE 16:0/22:5	58 ± 5		122 ± 12****
770,57	PC 18:1/18:1	108 ± 13		144 ± 19*
771,64	SM d22:0/18:1	7 ± 6		19 ± 4*
772,58	PC 18:1/18:0; PC 18:0/18:1; PC 20:1/16:0	415 ± 95		261 ± 72*
774,52	PS 17:1/18:0; PS 17:0/18:1	9 ± 2		5 ± 2*
776,56	PE O-18:1/22:5; PE P-18:0/22:5 PE O-18:0/22:6	171 ± 10		96 ± 10****
790,54	PE 18:0/22:6	1021 ± 79		610 ± 55***
794,57	PC 18:0/20:4; PC 16:0/22:4 PC 20:3/18:1	304 ± 10		233 ± 20***
797,65	SM d42:2	3 ± 4		16 ± 3**
834,53	PS 18:0/22:6; PS 22:6/18:0	356 ± 152		131 ± 21*
836,53	PC [42:11]; PE [44:11]; PS O-[38:3] PS P-[38:2]	0 ± 0		22 ± 16*
863,56	PI 18:1/18:0; PI 18:0/18:1	21 ± 5		32 ± 7*
885,55	PI 18:0/20:4	1720 ± 110		2480 ± 341**
909,55	PI 18:0/22:6; PI 20:2/20:4	40 ± 12		78 ± 19*
913,58	PI 18:0/22:4; PI 22:4/18:0 PI 20:0/20:4	10 ± 7		26 ± 6*

These data are the means ± S.E.M. of 4 retinal sections: an ANOVA was followed by a Bonferroni’s test or a Kruskal–Wallis test, and followed by Dunn’s test.

**Figure 4:**
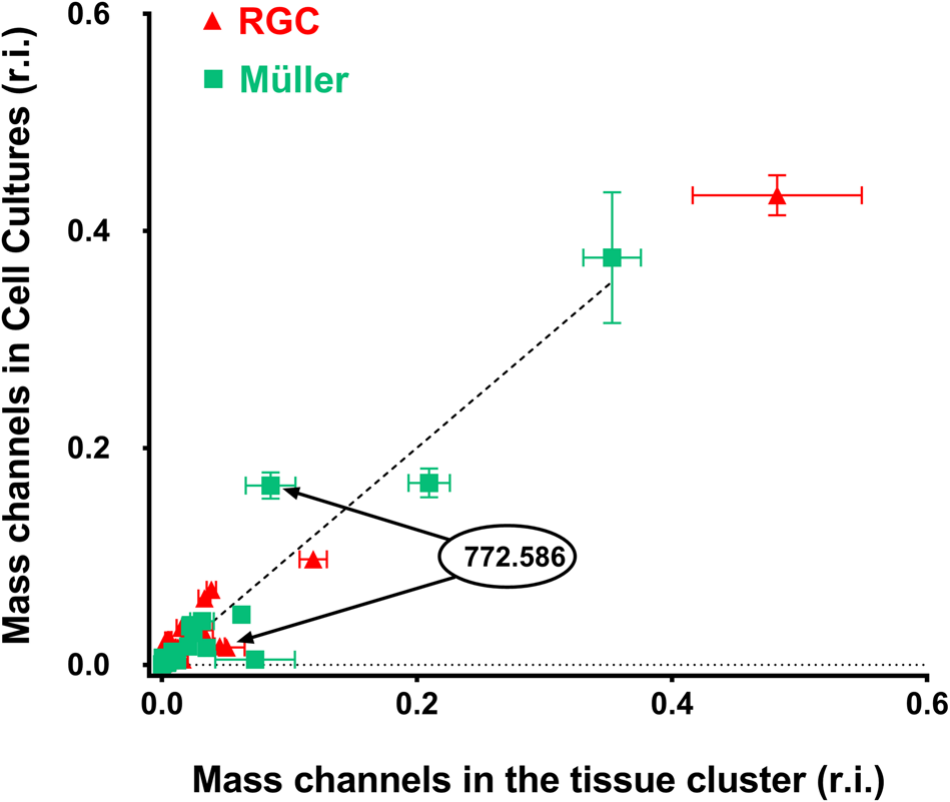
Correlation between the cell membranes and the tissue cluster spectra for RGC cells and Müller cells. (RGC Spearman r = 0.6992, p = 0.0001; Muller Spearman r = 0.7756, p < 0.0001).

To compare the results obtained from the Müller cells and RGCs in situ and in vitro, a correlation was made between the intensity of selected peaks obtained from retinal sections and their intensity in microarrays of membrane preparations. These data confirm that the results obtained in the sections were significantly correlated with those from the in vitro experiments (p < 0.0001, Fig. 4). A linear regression between the spectra from the RGC (Supplementary Fig. 2A) and Müller cells (Supplementary Fig. 2B) in the sections, and the in vitro spectra of RGCs and Müller cells always showed a stronger correlation between the spectra of the same cell type, irrespective of its origin (i.e.: sections or in vitro cultures). It was notable that the membrane correlation coefficient was stronger than that of the whole cells for both RGCs and Müller cells, such that the peaks obtained from the membrane preparations were more similar to the peaks obtained from the retinal sections (Supplementary Fig. 3). The distribution from the membranes in the microarrays was far more homogenous than that from the whole cells. This explained the weaker sensitivity and weaker signal/noise ratio in cells, due to the presence of fewer lipids per unit area.

Finally, a comparison of the lipid profiles obtained from the retinal sections and microarrays suggested a collection of six peaks (Table 3) that were characteristic of RGCs or Müller glia in both retinal sections and cultures. These lipids were identified by MS/MS, detecting characteristic fragmentation spectra for all the peaks (an example of the MALDI MS/MS assignment procedure is presented in Supplementary Fig. 4).

**Table 3:** Summary of the differential negative ions (*m/z*) selected and the corresponding lipid species, together with the relative intensity determined by MALDI-IMS analysis of retinal sections and cultures.

Ion	Molecular lipid species	Retinal sections		Retinal cultures				More intense in
		Intensity		Intensity				
		Müller	RGCs	Membranes		Cells		
				Müller	RGCs	Müller	RGCs	
794.57	PC 18:0/20:4; PC 16:0/22:4 PC 20:3/18:1	304 ± 10	233 ± 20	77 ± 9	37 ± 1	54 ± 21	52 ± 6	Müller cells
790.54	PE 18:0/22:6	1021 ± 79	610 ± 55	278 ± 22	211 ± 12	159 ± 65	165 ± 21	Müller cells
722.51	PE P-16:0/20:4	100 ± 29	163 ± 42	28 ± 5	69 ± 5	10 ± 10	43 ± 18	RGCs
764.52	PE 18:1/20:4; PE 16:0/22:5	58 ± 5	122 ± 12	7 ± 2	70 ± 5	2 ± 2	57 ± 10	RGCs
885.55	PI 18:0/20:4	1720 ± 110	2480 ± 341	622 ± 100	938 ± 40	103 ± 55	254 ± 54	RGCs
909.55	PI 18:0/22:6 PI 20:2/20:4	40 ± 12	78 ± 19	20 ± 3	74 ± 5	3 ± 2	21 ± 4	RGCs

The selected lipids represented are more abundant in Müller cells or RGCs, in both retinal sections and cultures.

## Discussion

Lipid biochemistry in the vertebrate retina is remarkable from many points of view, not least due to the specific accumulation of DHA (22:6) that is then esterified to different phospholipids and molecular species within the retina. The lipids of retinal membranes have been studied extensively and much information has been obtained^7^, however, this is the first time that in situ and in vitro techniques have been combined to evaluate the specific lipids accumulated by Müller glia and RGCs. There is evidence that certain lipids are distributed specifically in different retinal layers^33,36^. Given the importance of each of the different cell types in the retina, defining the lipids that might be specific to these individual cell types may provide unique information regarding the homeostatic state of this organ and the development of diseased states. This may be particularly true of the RGCs and Müller glia studied here, given their fundamental roles in preserving the homeostasis of the retina and in the transmission of visual information to the brain. After a successful correlation analysis whereby the results of in vitro experiments could be readily extrapolated to in situ results, a group of lipids were identified in which four peaks were more strongly represented in RGCs and two were more prevalent in Müller cells, both in sections and in microarrays.

The principal families of lipids in the rat retina are phosphatidylcholine (PC, ca. 40–50%), phosphatidylethanolamine (PE, ca. 30–35%), phosphatidylserine (PS, ca. 5–10%) and phosphatidylinositol (PI, ca. 3–6%), accounting for 85–90% of the total retinal phospholipids^42^. Therefore, it is not surprising that the peaks selected from the retinal samples were PCs, PEs and PIs. PCs, are usually the most abundant glycerophospholipids (GPLs) and the key building blocks of membrane bilayers. They have a small head group that can form hydrogen bonds through their ionizable amine group, which acts as a “chaperone” during membrane protein assembly and guides the folding of associated proteins. Four PCs were previously characterized in three distinct layers of a mouse retina section using MALDI-IMS, although none of them were specific to only one retinal layer: PC 16:0/16:0, PC 16:0/18:1, PC 16:0/22:6, and PC 18:0/22:6^30^. Here, one mass channel (794.5754 Da) was identified by MS/MS and MS^3^ that is contributed to by three PCs significantly more abundant in Müller cells than in RGCs, both in sections and cultures (PC 18:0/20:4; PC 16:0/22:4 and PC 20:3/18:1).

PEs are the second most abundant phospholipids in mammalian cells^43^, enriched in the inner side of membranes and particularly in the inner mitochondrial membrane. PEs are also enriched in arachidonic acid^44^ and they have quite notable activities: as a chaperone aiding membrane protein folding; in respiratory complexes; and in the initiation of autophagy^45^. Our results confirm that certain PEs are more abundant in RGCs than in Müller cells (PE P-16:0/20:4, PE 18:1/20:4 and PE 16:0/22:5). PE P-16:0/20:4 has been found in the mouse, rat and human brain^46^ and significantly, GPL signatures of retinal cells are similar to those of brain tissue^47^. Here, the PE P-16:0/20:4 was found in the retina and specifically, it was more abundant in RGCs than in Müller cells. PE 18:1/20:4 was previously seen to be present in both the optic nerve and retina, although the specific retinal cell type was not defined^33^. Moreover, with a high PE peak at 764 m/z corresponding to PE 18:1/20:4, this lipid might distinguish neural cells from non-neural cells^48^. According to our data and as found previously, PE 18:0/22:6 is more abundant in Müller glia and it is specifically located in the outer retinal layers where Müller cells may be found^33,49^.

PIs participate in essential metabolic processes, and they are primary sources of arachidonic acid and diacylglycerol. These second messengers serve as signaling molecules that regulate the activity of a group of related enzymes, known as protein kinase C, which regulates many crucial cell functions like proliferation, differentiation, metabolism and apoptosis. We detected two peaks (*m/z* 885.55 and 909.55) that correspond to three PIs more abundant in RGCs than in Müller cells, both in sections and microarrays. It is known that PIs are also main regulators of many ion channels and transporters, which are involved in neuronal excitability and synaptic transmission^50^. Thus, the more common representation of these lipids in RGCs than in Müller cells could be related to their neuronal activity. The basal peak at m/z 885.5 corresponded to PI 18:0/20:4, found in the nerve fiber/GC layer (by MALDI-IMS) and in the inner nuclear layer (INL) of the mouse and human retina^49^, and spreading into the outer plexiform layer (OPL)^36^ as well as the optic nerve, retina and sclera^33^. The *m/z* 909.5504 peak was identified as PI 18:0/22:6 and PI 20:2/20:4, PIs that are more commonly found in RGCs than Müller cells. However, in literature these lipids are not as common as PI 18:0/20:4 and to date, PI 18:0/22:6 has been found only in the cod retina^51^.

In summary, negative ion-mode imaging can be used to define the spatial distribution of a number of lipid species, including PEs, PCs and PIs, enabling us to carry out the first comparative study between in situ and in vitro assays. Combining different techniques that provided sufficiently high spatial resolution, distinguishing specific retinal cell layers, enabled the distributions of specific lipid to be defined. The fact that some lipids from the most relevant lipid families are more characteristic of RGCs or Müller cells suggests that they could fulfill roles in different cell activities. Interestingly, this technology could be used to compare healthy retinal tissue with pathological tissue in order to identify disease-related lipidomic changes in specific regions, such as advanced glycation and lipoxidation end products (AGEs and ALEs). Thus, further studies will provide more information on the implications of lipids in retinal diseases, identifying new therapeutic targets to slow or prevent disease progression.

## Methods

### Animals

Adult porcine eyes were obtained from a local abattoir and transported to the laboratory in cold CO_2_-independent Dulbecco’s modified Eagle’s medium (DMEM-CO_2_: Gibco-Life Technologies). The time between sacrifice and processing the eyes was 1 h. This study was carried out in strict accordance with the Guidelines for the Care and Use of Laboratory Animals from National Research Council (US). Moreover, all the experimental protocols complied with the European (2010/63/UE) and Spanish (RD53/2013) regulations regarding the protection of experimental animals, and they were approved by the Ethics Committee for Animal Welfare at the University of the Basque Country.

### Retinal cultures

RGCs and Müller glia were isolated from adult pig eyes to establish two types of culture: (1) RGC and (2) Müller cell cultures. The retinas were dissected out and dissociated enzymatically for 90 min with papain (Worthington Papain Dissociation kit, Worthington Biochemical Lakewood, NJ, USA) to obtain RGCs, or for 30 min to obtain Müller cells, according to the manufacturer’s instructions. The dissociated cells were recovered by centrifugation and the RGC cultures were prepared as described previously^52–55^. Briefly, the dissociated cells were passed through an ovomucoid inhibitor-albumin gradient, where more RGCs than Müller cells pass due to their smaller size (this step was excluded when preparing the Müller glia cultures). While this gradient does not purify RGCs to homogeneity, there is only minimal contamination of other cells. After purification, the cells were seeded in poly-l-lysine (10 µg/ml: Sigma–Aldrich, St. Louis, MO, USA) and laminin (10 µg/ml: Sigma–Aldrich, St. Louis, MO, USA) coated 6 well plates. The RGCs were seeded at 4 × 10^5^ viable cells per well and the pure Müller cell cultures were established at 1.2 × 10^7^ viable cells per well (as determined by trypan blue staining).

Different media were used for each culture type, yet both contained 1% l-glutamine (2 mM: Life Technologies, Carlsbad, CA, USA) and 0.1% gentamicin (50 mg/ml: Life Technologies, Carlsbad, CA, USA): Neurobasal-A medium supplemented with 2% B27 (Life Technologies, Carlsbad, CA, USA) for the RGC cultures; and DMEM with 10% FBS (Fetal Bovine Serum: Life Technologies, Carlsbad, CA, USA) for the pure Müller cell cultures. The different conditions were employed from the start of the culture and the medium was changed every 48 h. After 7 days in culture, the cells were trypsinized for 5 min at 37 °C, centrifuged, resuspended in phosphate buffered saline (PBS, pH 7.4) and left at 4 °C until use. At least 4 replicates of each culture type were performed in triplicate.

### Immunocytochemistry of cultures

In order to check the quality of the cultures, at least 3 wells of each culture were fixed in cold methanol and washed with PBS. After blocking non-specific antigens with blocking buffer (3% BSA and 0.1% Triton X-100 in PBS), the cells were incubated with the following primary antibodies at a dilution of 1:2000: a mouse anti-βIII-tubulin antibody as a specific RGC marker (RRID: AB_430874, Promega Madison, WI, USA); and as specific markers of Müller glia, a rabbit anti-Vimentin antiserum (RRID: AB_1524552, Abcam, Cambridge, England), a rabbit anti-p75NTR antiserum (RRID: AB_2728801, Abcam, Cambridge, England), a rabbit anti-CRALBP antiserum (RRID: AB_1658655, Abcam, Cambridge, England) and a rabbit anti-Glutamine synthetase antiserum (RRID: AB_302521, Abcam, Cambridge, England). After washing the cells, antibody binding was detected with an anti-mouse Alexa Fluor 488 or an anti-rabbit Alexa Fluor 555 (Life Technologies, Carlsbad, AC, USA) secondary antibody, diluted 1:1000. In addition, the cell nuclei were labeled with DAPI (Life Technologies, Carlsbad, AC, USA) at a dilution of 1:10,000.

### Cell membrane isolation

The cells isolated from pig Müller glia or RGC cultures were homogenized using a Teflon-glass grinder (Heidolph RZR 2020) and a disperser (Ultra-Turrax^®^ T10 basic, IKA) in 50 mM Tris buffer (TB) supplemented with 1 mM EGTA, 3 mM MgCl_2_ and 250 mM sucrose. The crude homogenates were centrifuged at 40 × *g* for 5 min and the resulting supernatants were centrifuged again at 18,000 × *g* for 15 min at 4 °C (Microfuge^®^ 22R centrifuge, Beckman Coulter). The pellets were washed in 20 volumes of 50 mM TB and re-centrifuged under the same conditions. The protein concentration of each sample was measured using the Bradford method and aliquots of the homogenate were stored at − 80 °C until use.

### Microarray development

Microarrays were established from whole cells or membrane preparations printed onto glass slides using a non-contact microarrayer (Nano_plotter NP 2.1) and a printing solution^55^. The piezoelectric tips dispense 4 nl per spot, printing each sample in triplicate. These microarrays were stored at − 20 °C until use.

### Tissue collection for retinal sections

Adult eyes were enucleated, removing the cornea, crystalline lens and vitreous humor. The retina was then carefully separated from the rest of the eye, cut into small rectangular pieces and folded back over itself like a closed book or “sandwich” to maintain the RGCs internally when the tissue was then frozen at – 20 °C. The use of this “sandwich” aimed to protect the layer of ganglion cells at the edge of the section and to avoid interactions of the OCT with the matrix used. In this way, we could also visualize a duplicate image of the retina in each section (Fig. 3). The retinal “sandwich” was then partially embedded in OCT medium to obtain cryosections of the retinal aspect and not of the OCT embedded tissue (14 μm thick). These sections were then stored at − 80 °C in an atmosphere of N_2_ to protect them from oxygen and moisture degradation.

### Sample preparation for IMS

Retinal sections and microarrays were thawed, and DAN (1,5-Diaminonaphthalene)^57^ was deposited on them using a glass sublimator (Ace Glass 8023)^58^. The matrix formed a uniform thin layer that enables retinal sections and microarrays to be scanned in negative ion mode for several hours. Only negative-ion mode was employed in this study as a wider variety of lipid species can be detected in this way, whereas PC species are those predominantly detected in positive-ion mode. For example, plasmalogens are usually obscured by PCs in positive-ion mode. Likewise, sulfatides are detected in negative-ion mode, and any interference between PC and PE is avoided. This analysis was performed as described previously^59^, whereby a LTQ-Orbitrap XL analyzer (ThermoFisher, San Jose, CA, USA) equipped with an N2 laser (100 µJ max power, elliptical spot, 60 Hz repetition rate) scanned the material in negative ion mode at a spatial resolution between 15 and 25 µm. A mass resolution of 60,000 and 100,000 was used to record the data from the full scan spectra, and that of around 2000 for MS/MS and MS^3^ due to the higher sensitivity of the Ion Trap (IT). The scanning range in full scan spectra was 550–1200.

### Immunohistochemistry of retinal sections

After the sections were scanned they were washed with methanol at room temperature (RT) for 5 min to remove the DAN and they were then fixed with 4% PFA (paraformaldehyde) at RT for 2 min. The sections were immunostained as described previously^38^ and after washing twice in PBS-Triton X-100 for 10 min, they were incubated overnight with the primary antibodies (diluted 1:2000): a mouse anti-βIII-tubulin antibody (Promega Madison, WI, USA) as a specific RGC marker; and a rabbit anti-Vimentin antiserum (Abcam, Cambridge, England) as a specific marker of Müller glia. After washing twice in PBS, antibody binding was detected for 1 h with an Alexa Fluor 555 conjugated goat anti-mouse antibody (Invitrogen, Eugene, Oregon, USA) and an Alexa Fluor 488 conjugated goat anti-rabbit antibody (Invitrogen, Eugene, Oregon, USA), both diluted 1:1000 in PBS-BSA (1%). Finally, the sections were washed twice with PBS for 10 min and mounted with a coverslip in PBS-Glycerol (1:1).

### Data and statistical analysis

The spectra acquired were aligned to maximize the correlation with the overall averaged spectrum and normalized using dedicated software (MSI Analyst, Noray Bioinformatics S.L.). During parsing, the size of the data was reduced to eliminate all the peaks whose intensity was lower than 0.5% of the strongest peak on the spectrum and the spectra were normalized using a total ion current algorithm^60^. The spectra were also aligned using the Xiong method^61^ assuming a maximum misalignment of 0.02 a.m.u., very conservative for an orbitrap analyzer. For graphical representation, no interpolation or smoothing algorithms and no de-noising procedures were used, always trying to maintain the original aspect of the data. Re-scaling was required for some images reflecting the distribution of the very weak peaks.

Statistical analysis to identify the different areas in the sections was carried out using MATLAB (MathWorks, Natick, USA), employing divisive hierarchical clustering with Rank Compete^62^ and k-means^63^. After determining the cluster of Müller cells and RGCs, a principal component analysis (PCA)^64^ and ANOVA were used to identify the specific lipids for each cell type in the retinal sections and microarrays.

### Peak assignment

The peaks of interest were identified directly by MALDI-MS/MS and MS^3^ on the tissue samples in order to discriminate chemical variants of the lipids with identical numbers of acyclic carbons and double bonds. To perform fragmentation we used collision-induced dissociation (CID) and an ion trap to detect the fragments, a set-up that improves the sensitivity for the fragments with little cost of losing mass resolution. The mass window of the ion trap allows is approximately one Da wide and thus, we could identify fragments that belonged to our parent masses and those belonging to other species close to the parent masses (see Supplementary Fig. 4 and Supplementary Table 1). The assignment of lipid species was facilitated by a database created using tissue-dependent lipid compositions. The database was calculated in silico by combining fatty acids and head groups, and with the aid of the Lipid Maps database (https://www.lipidmaps.org/). The experimental values of the precursor molecules, and of the fragments after MS/MS and MS^3^ analysis were compared with this database, and with the Lipid MAPS and Madison Metabolomics Consortium (https://mmcd.nmrfam.wisc.edu/) databases, using 0.005 Da as a window of tolerance for the full scan precursors in order to identify each mass channel.

## Supplementary information

Supplementary Information 1.

Supplementary Information 2.
