# Supplementary material for: Comparative lipidomic analysis of mammalian retinal ganglion cells and Müller glia in situ and in vitro using High-Resolution Imaging Mass Spectrometry
Source: Sci Rep. 2020 Nov 18;10:20053. doi: 10.1038/s41598-020-77087-x (PMC7674471; doi:10.1038/s41598-020-77087-x)
Supplement: Supplementary file 1 — Supplementary Information 1. [file 41598_2020_77087_MOESM1_ESM.pdf]

Supplementary information

Comparative lipidomic analysis of mammalian retinal ganglion cells and Müller glia *in situ* and *in vitro* using High-Resolution Imaging Mass Spectrometry

Xandra Pereiro[*1], Roberto Fernández[2,3], Gabriel Barreda-Gómez[3], Noelia Ruzafa[1], Arantxa Acera[1], Javier Araiz[4], Egoitz Astigarraga[3] and Elena Vecino[1]

*Correspondence to: xandra.pereiro@ehu.eus

[1]Experimental Ophthalmic-Biology Group (GOBE), Dept. of Cell Biology and Histology, University of Basque Country (UPV/EHU), Leioa, Vizcaya, Spain.

[2]Dept. of Physical Chemistry, University of the Basque Country (UPV/EHU), Leioa, Vizcaya, Spain.

[3]IMG Pharma, Derio, Vizcaya, Spain.

[4]Dept. Ophthalmology, University of the Basque Country (UPV/EHU), Leioa, Vizcaya, Spain.

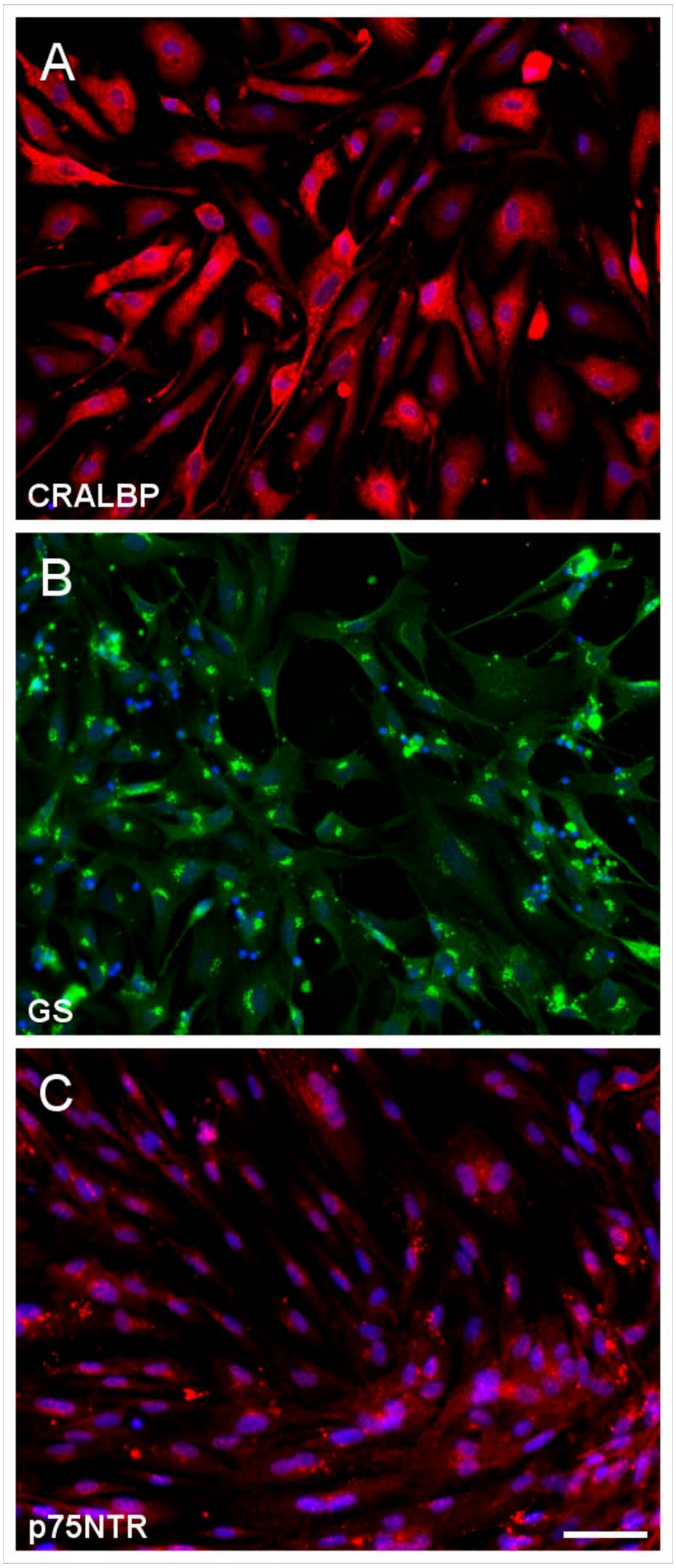

Supplementary Figure 1. Expression of Müller cell markers in adult pig Müller cell cultures. Images from Müller cells labelled with antibodies against CRALBP (red, A), glutamine synthetase (GS, green, B), p75NTR (red, C) and DAPI in blue. Scale bar, 50 µm.

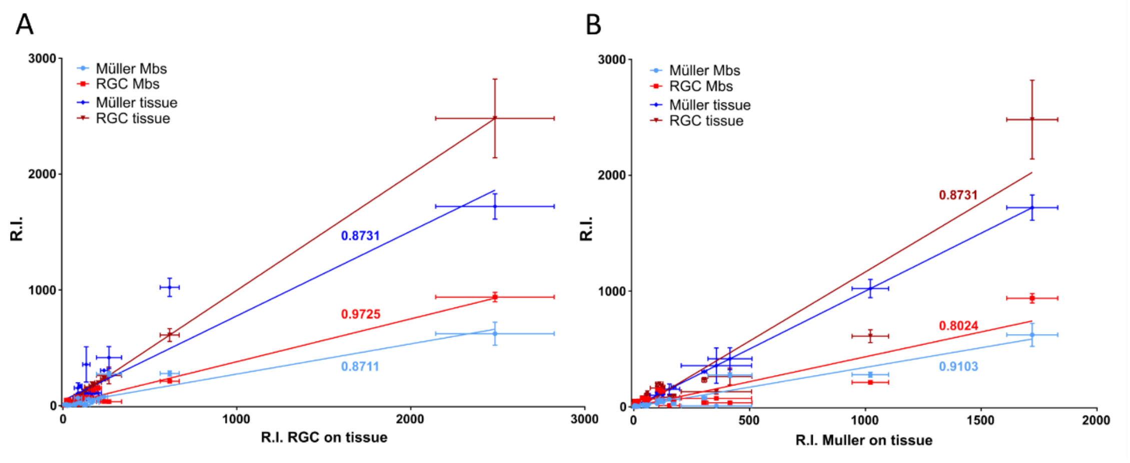

Supplementary Figure 2. Linear regression and R^2 between A) RGC in section cluster spectra against: RGC in section cluster spectra, Muller in section cluster spectra, RGC cell membranes and Müller cell membranes B) Müller in section cluster spectra against RGC in section cluster spectra, Muller in section cluster spectra, RGC cell membranes and Müller cell membranes. In both cases, the correlation is better between the cells and tissue of the same cell type than between cells and cells or tissue and tissue.

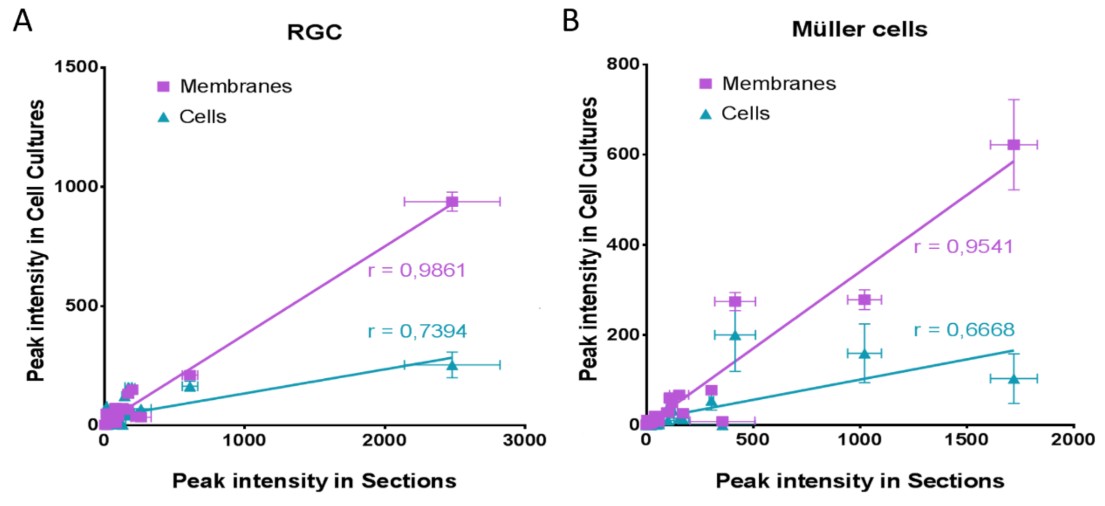

Supplementary Figure 3. Linear regression between cell culture spectra, membrane homogenates and whole cells, and in section spectra of RGC (A) and Müller cells (B).

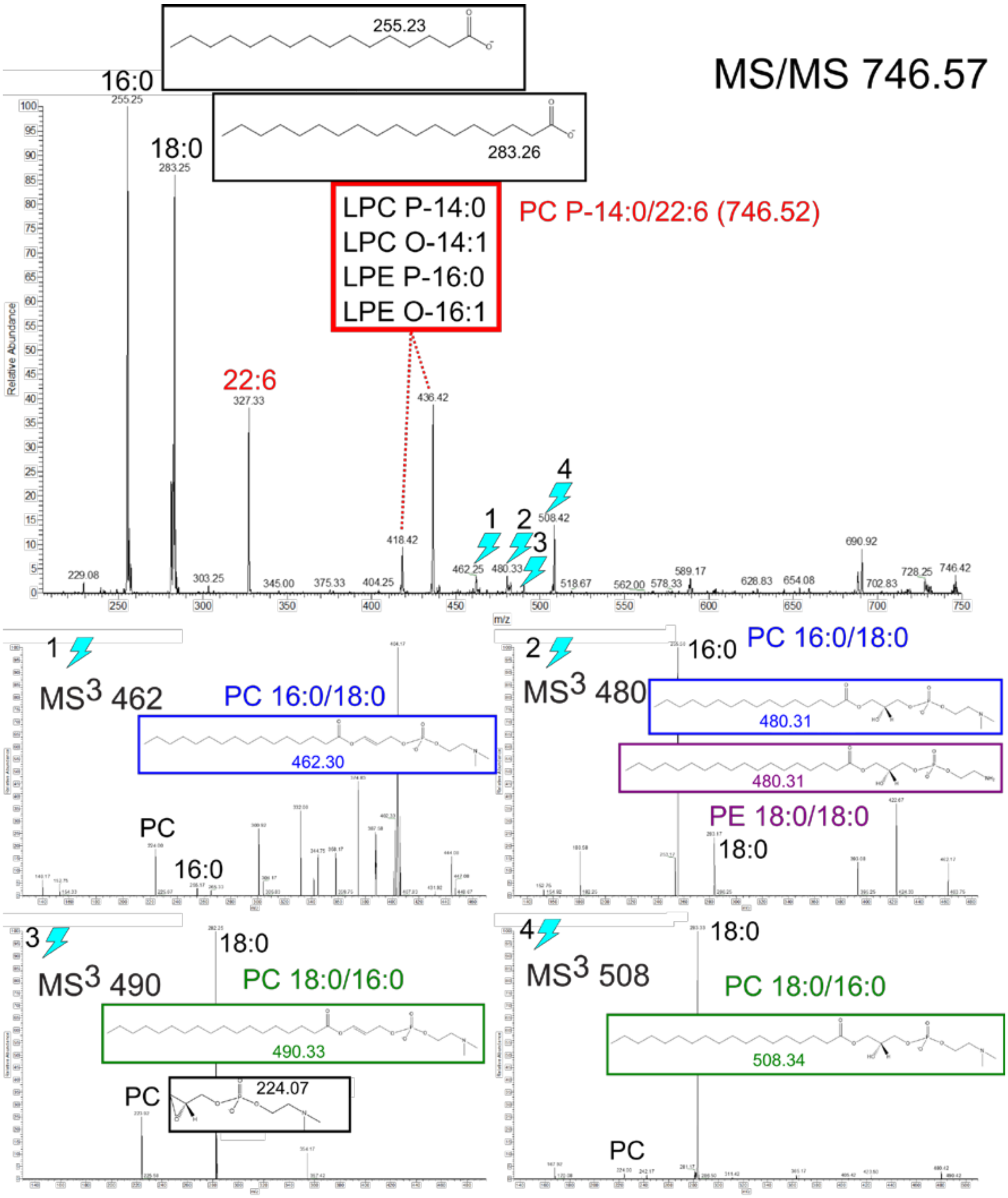

Supplementary Figure 4. On the top of the figure the MS/MS spectrum of the peak 746.57, on the bottom the MS3 of the four peaks selected in the MS/MS spectrum. In red, fragments of a species with different monoisotopic mass but in the fragmentation window of 1 Da around 746.57. In green, blue and purple, species confirmed by fragmentation of the mass channel 746.57.

Supplementary Table 1. MS/MS, MS^3 and MS^4 fragments detected and identified for each parent mass. In red, those species and its fragments that are in the same fragmentation window but not in the exact monoisotopic mass of the peak we want to identify.

599,32		Lyso-PI 18:0; PI O-16:0/2:0							
	MS2	283 Da (18:0)	241 Da (O-16:0)						
700,52		PE P-16:0/18:1; PE O-16:1/18:1							
	MS2	281 Da (18:1)	255 Da (16:0)	436 Da (PE O-16:1; PE P-16:0)	418 Da				
				196 Da (PE)					
	MS3			239 Da (O-16:1; P-16:0)					
716,52		PC 16:1/16:0; PC 16:0/16:1; PE 18:1/16:0; PE 16:0/18:1; PE 18:0/16:1							
	MS2	255 Da (16:0)	281 Da (18:1)	253 Da (16:1)	452 Da	478 Da	480 Da	450 Da	
	MS3				255 Da (PE 16:0)	281 Da (18:1) (PE)	255 Da (16:0) (PC)	196 Da (PE)	
	MS3					253 Da (16:1) (PC)	283 Da (18:0) (PE)		
722,51		PE P-16:0/20:4; PE O-16:1/20:4							
	MS2	303 Da (20:4)	255 Da (16:0)	436 Da	418 Da				
	MS3			196 Da (PE)					
	MS3			239 Da (O-16:1; P-16:0)					
726,54		PE O-18:2/18:1; PE P-18:1/18:1							
	MS2	281 Da (18:1)	255 Da (16:0)	462 Da	444 Da				
	MS3			196 Da (PE)					
	MS3			265 Da (O-18:2;P-18:1)					
738,50		PE 16:0/20:4							
	MS2	303 Da (20:4)	255 Da (16:0)	452 Da					
	MS3			255 Da (16:0)					
	MS3			196 Da (PE)					
740,52		PE 18:1/18:2; PE 16:0/20:3							

	MS2	279 Da (18:2)	281 Da (18:1)	305 Da (20:3)	255 Da (16:0)	241 Da (O-16:0)	456 Da	478 Da	
	MS3						241 Da (O-16:0)	281 Da (18:1)	
	MS3							196 Da (PE)	
742,54		PC 18:2/16:0; PE 18:1/18:1							
	MS2	281 Da (18:1)	255 Da (16:0)	478 Da	504 Da	460 Da			
	MS3			281 Da (18:1)	279 Da (18:2)				
	MS3								
746,57		PC 18:0/16:0; PC 16:0/18:0; PE 18:0/18:0; PC P-14:0/22:6 (746.52)							
	MS2	255 Da (16:0)	283 Da (18:0)	327 Da (22:6)	436 Da	508 Da	480 Da	462 Da	490 Da
	MS3					283 Da (18:0) (PC)	255 Da (16:0) (PC)	224 Da (PC)	283 Da (18:0)
	MS3						283 Da (18:0) (PE)		224 Da (PC)
764,52		PE 18:1/20:4; PE 16:0/22:5							
	MS2	303 Da (20:4)	281 Da (18:1)	329 Da (22:5)	255 Da (16:0)	478 Da	452 Da	526 Da	434 Da
	MS3					281 Da (18:1) (PE)	255 Da (16:0) (PE)	329 Da (22:5)	255 Da (16:0) (PE)
	MS3					196 Da (PE)			
770,57		PC 18:1/18:1							
	MS2	281 Da (18:1)	506 Da						
	MS3		281 Da (18:1) (PC)						
	MS3		224 Da (PC)						
771,64		SM d22:0/18:1							
	MS2	281 Da (18:1)	507 Da (SM d22:0)						
772,58		PC 18:1/18:0; PC 18:0/18:1; PC 20:1/16:0							
	MS2	281 Da (18:1)	283 Da (18:0)	255 Da (16:0)	309 Da (20:1)	327 Da (22:6)	508 Da	506 Da	534 Da
	MS3						283 Da (18:0) (PC)	281 Da (18:1) (PC)	309 Da (20:1) (PC)
	MS3						224 Da (PC)		224 Da (PC)
774,52		PS 17:1/18:0; PS 17:0/18:1; PC O-16:1/22:6 (774,54); PE O-18:1/22:6 (774,54)							

	MS2	327 Da (22:6)	283 Da (18:0)	464 Da	446 Da	687 Da (-serine)			
	MS3					405 Da			
	MS4					267 Da (17:1)			
	MS4					269 Da (17:0)			
776,56		PE O-18:1/22:5; PE P-18:0/22:5; PE O-18:0/22:6							
	MS2	329 Da (22:5)	327 Da (22:6)	283 Da (18:0)	255 Da (16:0)	466 Da	464 Da	446 Da	444 Da
	MS3					196 Da (PE)	196 Da (PE)		
	MS3					269 (17:0; O-18:0)	267 (17:1; O-18:1; P-18:0)		
790,54		PE 18:0/22:6; PE 22:6/18:0; PS O-16:1/22:6 (790.50)(+Na-2H)							
	MS2	283 Da (18:0)	327 Da (22:6)	480 Da	506 Da	462 Da	524 Da		
	MS3			283 Da (18:0) (PE)	196 Da (PE)	196 Da (PE)	283 Da (18:0) (PS)		
	MS3			196 Da (PE)			327 Da (22:6) (PE)		
794,57		PC 18:0/20:4; PC 16:0/22:4; PC 20:3/18:1; PE 22:4/18:0							
	MS2	331 Da (22:4)	283 Da (18:0)	303 Da (20:4)	255 Da (16:0)	480 Da	508 Da	530 Da	556 Da
	MS3					283 Da (18:0) (PE)	283 Da (18:0) (PC)	305 Da (20:3)	331 Da (22:4) (PE)
	MS3					255 Da (16:0) (PC)	224 Da (PC)		
	MS3					196 Da (PE)			
797,65		SM d22:2/20:0							
	MS2	303 Da (20:4)	283 Da (18:0)	283 Da (18:0)	511 Da	533 Da	467 Da	485 Da (SM d22:2)	
	MS3				283 Da (18:0) (PG)	307 Da (20:2)	331 Da (22:4)		
	MS3				255 Da (16:0)				
834,53		PS 18:0/22:6; PS 22:6/18:0							
	MS2	283 Da (18:0)	747 Da (-Serine)						
	MS3		283 Da (18:0)						
	MS3		327 Da (22:6)						
	MS3		419 Da (PA 18:0)						

	MS3		463 Da (PA 22:6)						
	MS3		437 Da						
836,53		PC [42:11]; PE [44:11]; PS O-[38:3]; PS P-[38:2]							
	MS2	749 Da (-Serine)	281 Da (18:1)	778 Da	283 Da (18:0)	331 Da (22:4)	241 Da (15:0)	297 Da (19:0)	580 Da
	MS3								297 Da (19:0/O-20:0)
863,56		PI 18:1/18:0; PI 18:0/18:1							
	MS2	283 Da (18:0)	281 Da (18:1)	581 Da (PI 18:0)	579 Da (PI 18:1)				
885,55		PI 18:0/20:4; PI 20:4/18:0							
	MS2	283 Da (18:0)	303 Da (20:4)	581 Da (PI 18:0)	601 Da (PI 20:4)				
909,55		PI 18:0/22:6; PI 22:6/18:0; PI 20:2/20:4							
	MS2	283 Da (18:0)	297 Da (19:0; O-20:0)	327 Da (22:6)	581 Da (PI 18:0)	625 Da (PI 22:6)	303 Da (20:4)	605 Da (PI 20:2)	
913,58		PI 18:0/22:4; PI 22:4/18:0; PI 20:0/20:4							
	MS2	283 Da (18:0)	331 Da (22:4)	303 Da (20:4)	581 Da (PI 18:0)	629 Da (PI 22:4)	609 Da (PI 20:0)		

* PC ionization [M-CH3]-; SM ionization [M-CH3]-; PE ionization [M-H]-; PI ionization [M-H]-; PS ionization [M-H]-

** in red, other species which are inside the fragmentation mass window but not at the same monoisotopic mass

*** in orange, specie we weren't able to confirm by fragmentation

Supplementary Data 1. Raw data from the clusters corresponding to the Müller cells and RGC areas in the sections.
